# Two cases of Prune Belly Syndrome from Kagera Region Tanzania

**DOI:** 10.24248/eahrj.v4i1.630

**Published:** 2020-06-26

**Authors:** Jonas P. Kessy, Rune N. Philemon, Ben C. Hamel

**Affiliations:** a Bukoba Regional Referral Hospital, Paediatric and Child Health Department, Bukoba, Tanzania; b Kilimanjaro Christian Medical University College, Moshi, Tanzania; c Department of Human Genetics, Radboud University Medical Center, Nijmegen, The Netherlands

## Abstract

Prune Belly Syndrome is a rare congenital disorder with unknown aetiology, consisting of a triad of abdominal muscle wall weakness, undescended testes, and urinary tract abnormalities. We are unaware of any preceding report of Prune Belly Syndrome in Tanzania, and here we describe two cases reported in Kagera region. The first case is a 2 month old boy with the triad of Prune Belly Syndrome along with pectus carinatum who died due to septicaemia. This case posed a diagnostic challenge at birth and during the natal period. Paucity of comprehensive knowledge of congenital malformations at the peripheral health facilities may have also contributed to the diagnostic challenge in the first place.

The second case is a neonate who was referred to regional referral hospital where he was diagnosed with Prune Belly Syndrome at the age of four weeks. Because of limited capacity to manage congenital malformations at the regional referral hospital, he was referred to an urologist at the zonal referral hospital. However, inadequacies in supporting systems to the parents compounded care of the neonate with Prune Belly Syndrome.

High index of Prune Belly Syndrome suspicion is needed in a resource limited setting in order to timely make diagnosis. There is also a need to strengthen institutional and individual's capacity for prenatal screening to detect congenital anomalies at an early stage of foetus development. Multidisciplinary management approach is necessary in order to improve the quality of life for patients with Prune Belly Syndrome. Psychosocial and medical support systems should be put in place in order to enhance preparedness for patient care in resource limited settings including equipping the referral hospital with different specialists and ensuring availability of basic investigations for patients.

## BACKGROUND

Prune Belly Syndrome (PBS) is a rare congenital disorder characterized by a triad of deficient abdominal wall muscles, cryptorchidism and urinary tract anomalies (including hydroureteronephrosis). The syndrome affects 3.8 per 100,000 live male births^1–4^. Over 95% of patients are boys^5^. Some studies have suggested that the abdominal muscle deficiency is due to foetal abdominal distension of different causes including urethral obstruction with enlarged bladder and hydronephrosis. Also mesenchymal developmental defects have been suggested as the underlying defect^1^.

The cause of PBS is unknown^6^. Familial case reports and the higher incidence in males have suggested involvement of an X linked factor and recently mutations in the X linked gene *Filamin A* were reported^7^. However, in other cases an autosomal recessive mode of inheritance (OMIM #100100; *CHRM3* gene) has been suggested^5^.

Hydronephrosis and hydroureters have been found as features associated with PBS through abdominal ultrasound in 53.3% of cases^5^.

There are few reports from developing countries regarding the pattern of renal involvement and management outcome of patients with PBS. Lack of sufficient follow up to determine the course of the disorder may explain the existence of few PBS reports in developing countries^8^. Hydronephrosis and oligohydramnios found during antenatal care should raise suspicion of PBS and followed by systematic examination and regular prenatal follow up^1^. We report two cases with clinical manifestations associated with PBS, including deficient abdominal wall muscles, hydronephrosis and cryptorchidism.

### Ethical Considerations

Permission to publish cases of two children with PBS was obtained from Regional Medical Officer. However, final decision was made by parents who consented for the publication of the case reports and any complementary image. Parents were informed that the identity of the child will be kept confidential

### Case Presentations

#### Case 1:

A 2 month old boy from Kagera region, located in the Northwestern corner of Tanzania on the western shore of Lake Victoria, was admitted at Bukoba Regional Referral Hospital (BRRH) on 27^th^ July 2018 as a referral case from Kabale dispensary in Bukoba district. Bukoba Regional Referral Hospital is owned by the Government with the capacity of 308 beds, of which 60 are for paediatric patients and 50 for surgical cases. At a time of admission, the mother reported that the child had fever, cough and difficulty in breathing for the past 3 days. He had no vomiting, no diarrhoea, and was passing urine normally. He had received 3 days of antibiotics prior to admission and was referred from Kabale dispensary to Bukoba Regional Referral Hospital following deterioration of his condition. Patient past medical history, family history and his mother's pregnancy history did not contribute to diagnosis of PBS. He is the second born in a family with no history of PBS symptoms.

**FIGURE 1.**
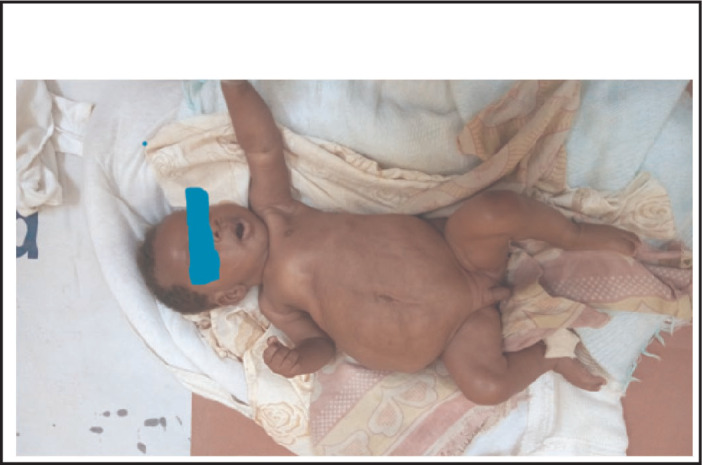


**FIGURE 2.**
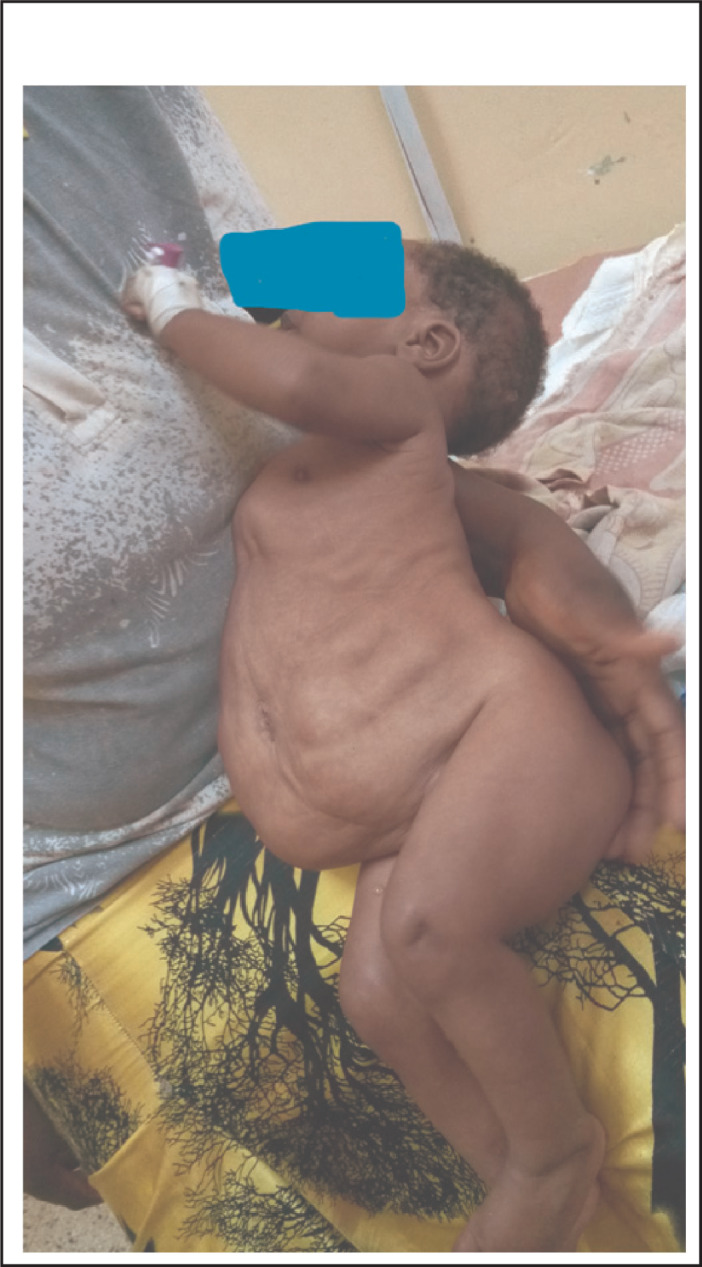


**FIGURE 3.**
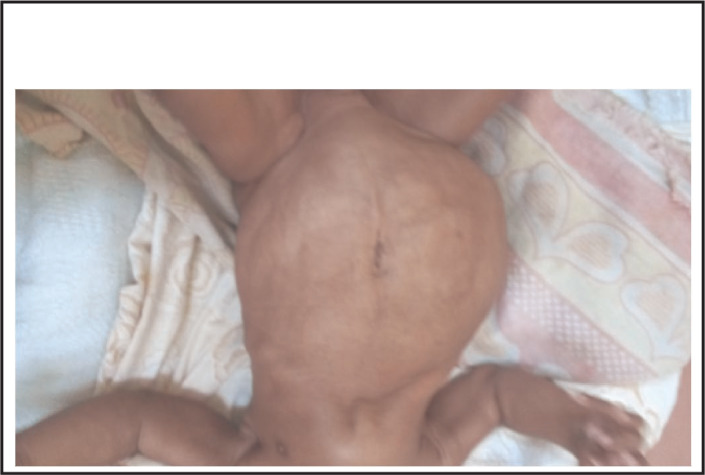


**FIGURE 4.**
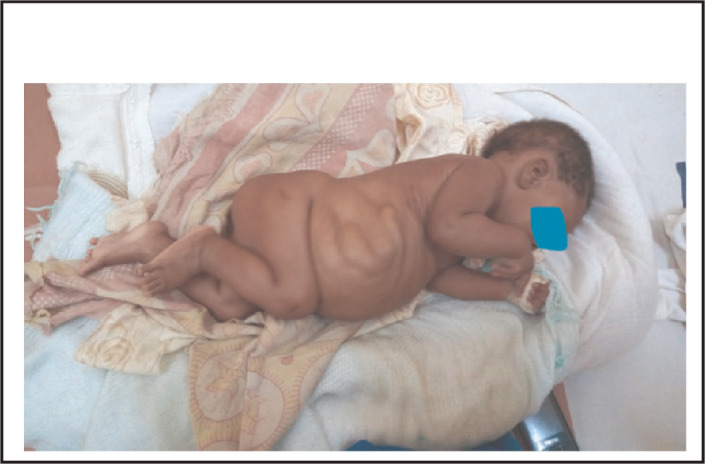


On physical examination his body temperature was 38.5 °C with an increased pulse rate and respiratory rate. His blood pressure was 105/65 mmHg. His weight for length was between median and -1SD. Abdominal examination revealed a floppy abdomen with poorly developed muscles, asymmetric with absent rectus abdominis muscles. There was no palpable mass, and his abdomen was soft. Both testes were undescended (Figure 1-4). Other systems were unremarkable.

**FIGURE 5.**
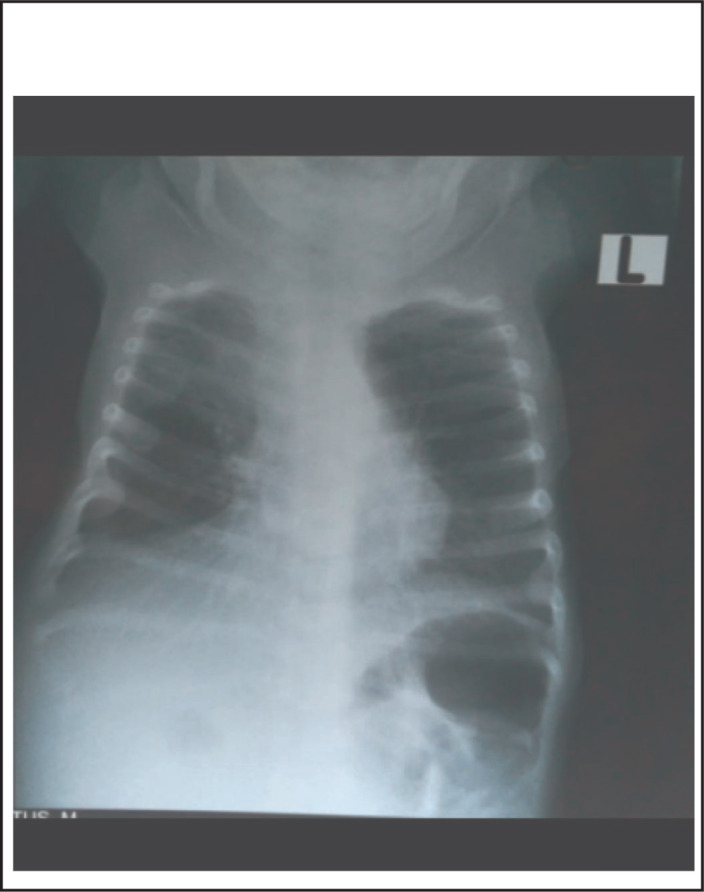


**FIGURE 6.**
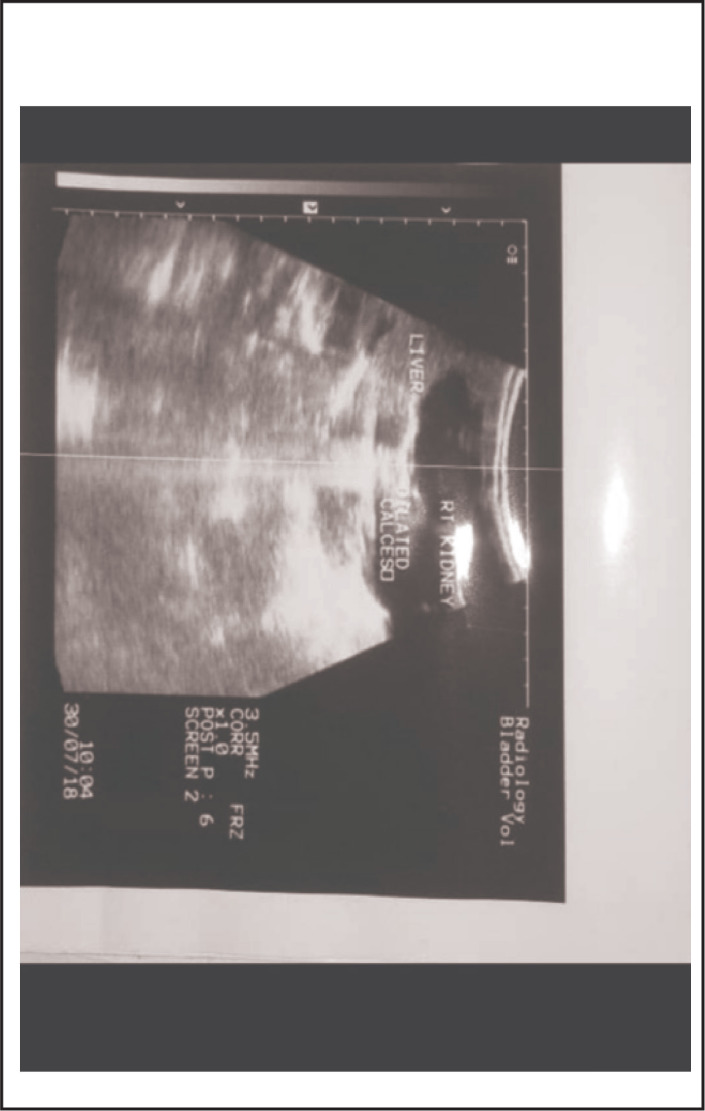


Laboratory investigations (Table 1) showed leukocytosis, predominantly of lymphocytes, microcytic hypochromic anaemia and almost normal creatinine. Chest x-ray showed opacities in the right upper lobe, suggestive of right upper lobe pneumonia, and enlarged costal-chondral junctions suggestive of rickets (Figure 5). Abdominal ultrasound showed bilateral hydronephrosis (Figure 6). After investigations, the child was treated as a case of severe pneumonia, improved after seven days of antibiotics (ampicillin and gentamicin) and was discharged with iron supplements and cod liver oil. His parents were also counselled about PBS and the care they can give him at home. He was given an appointment to come back for follow up after one month and referred to Bugando zonal referral hospital in Mwanza for surgical consultation and urology review, but he never got there. However, the patient was not seen till three months later when he was brought with complaints of diarrhoea, vomiting and fever for two weeks for which he been receiving - had been receiving treatment at Kabale dispensary without improvement. On arrival, the child was severely ill, restless, had sunken eyes and skin pinch went back slowly. His temperature was 39.3°C, with increased pulse rate and respiratory rate and peripheral capillary oxygen saturation (SpO_2_) of 92% on air. Other systems were normal. Laboratory investigations (Table 1) showed severe leukocytosis and granulocytosis with microcytic anaemia with low red blood cells (RBC). He was given intravenously administered antibiotics (Ceftriaxone) for treatment of suspected septicaemia, intravenous fluid to correct the hydration status, paediatric zinc, blood transfusion (140mls), and oxygen therapy. The child's condition continued to deteriorate and on the sixth day of admission he died.

**TABLE 1.** Laboratory Results

Investigation	Case 1		Case 2	Normal values
	On admission (27/07/2018)	On re-admission (26/11/2018)	At OPD (27/09/2019)	
Creatinine	49.0 µmol/L		134.0 µmol/L.	53-110 µmol/L9.
WBC	16.2 x 103/mm^3^	39.2 x 103/mm^3^	8.1 x 103/mm^3^	2.0 – 12.3 x 103/mm^3^
Lymphocytes	66.8,%	9.4%	78.6%	20.0 – 40.0%
Granulocytes	24.9%	87.4%	7.1%	26.0 - 79.0%
Monocytes	8.3%	3.2%	14.3%	2.0 – 10.0%
Hb	9.9g/dl	7.3g/dl	13.8g/dl	11.0 – 16.5g/dl
MCV	75 µm^3^	69 µm^3^	87 µm3	80-100 µm^3^
MCH	20.0/pg	23.0 pg	29.4 pg	26.0 - 34.0pg
Platelets	228 x 103/mm^3^	438 x 103/mm^3^	315 x 103/mm^3^	145 - 450 x 103/mm^3^
RBC	4.98 x 106/mm^3^	3.15 x 106/mm^3^	4.7 x 106/mm^3^	4.70 – 5.63 x 106/mm^3^

#### Case 2:

The second case is a neonate of 4 weeks, who came as a referral case from Rwamishenyi health centre in Bukoba Municipality on 27^th^ September 2019 with suspicion of intestinal obstruction. On reviewing the patient at the paediatric clinic, his parent reported that they noted abdominal swelling on the left side since birth. He had no vomiting, no diarrhoea, no history of fever, no cough and was passing urine normally. He was breast feeding well and had received immunization, oral polio vaccine and Bacillus Calmette-Guerin (BCG) vaccine at birth. His prenatal, natal, postnatal and pregnancy history were not contributory. He was the first born in the family. There were no similar cases known in the family.

**FIGURE 7.**
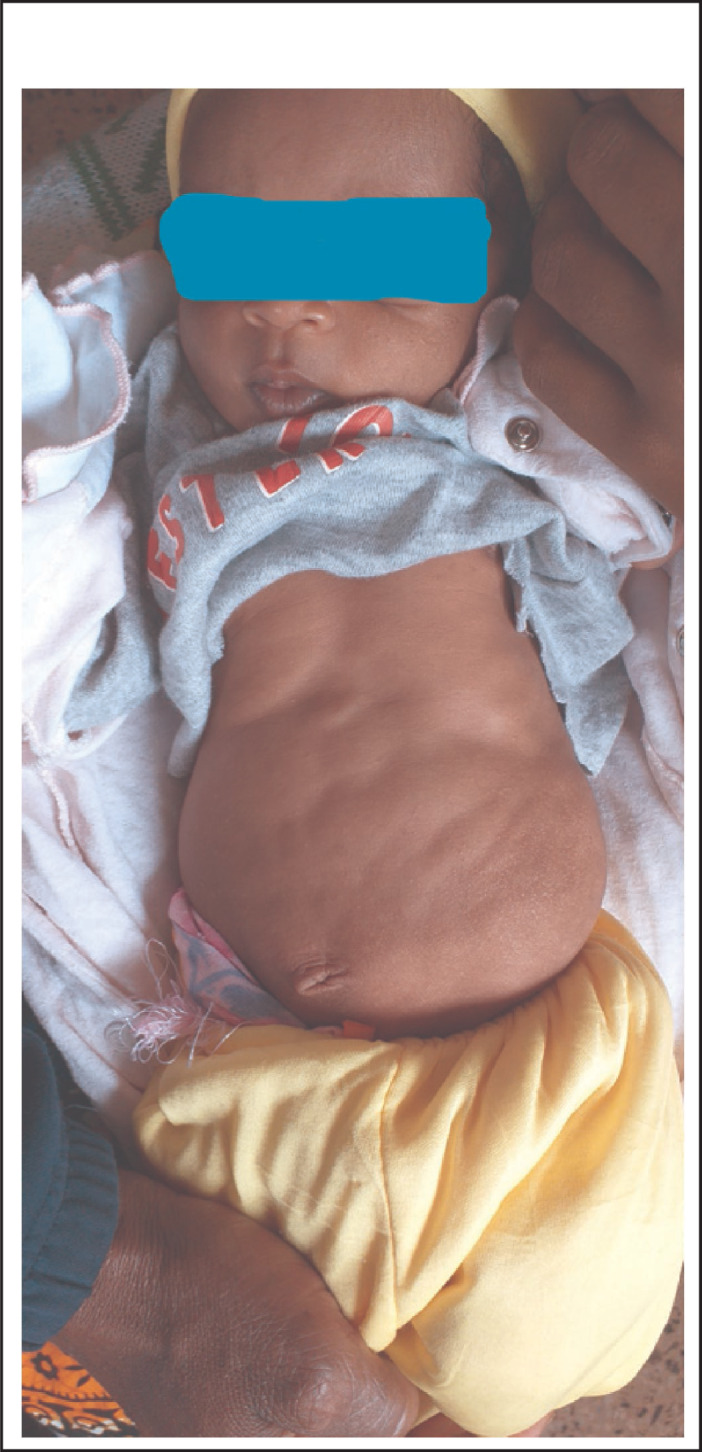


On physical examination a pulse rate (PR) of 148 beats/min, respiratory rate (RR) of 55 breaths/min and a body temperature (T) of 36.4 °C were noted. Abdominal examination revealed a flabby abdomen with poorly developed muscles, asymmetric, with absent rectus abdominis muscles with a soft mass on the left side of the abdomen. Both testes were undescended. On examination of the chest, pectus excavatum was noted (Figure 7 **to** 8) as well as normal vesicular breathing sounds. Other systems were unremarkable.

**FIGURE 8.**
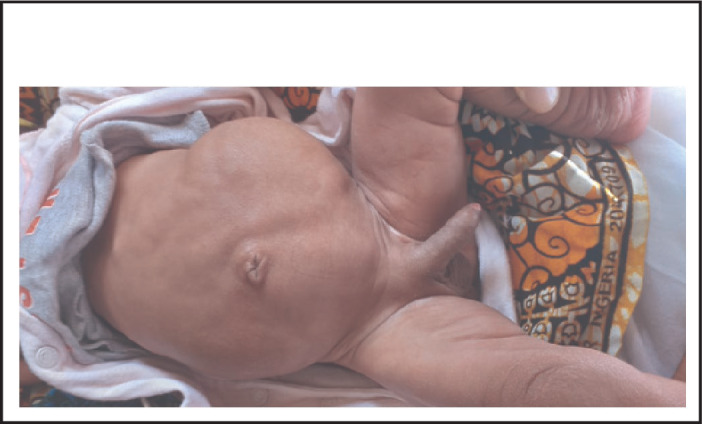


**FIGURE 9.**
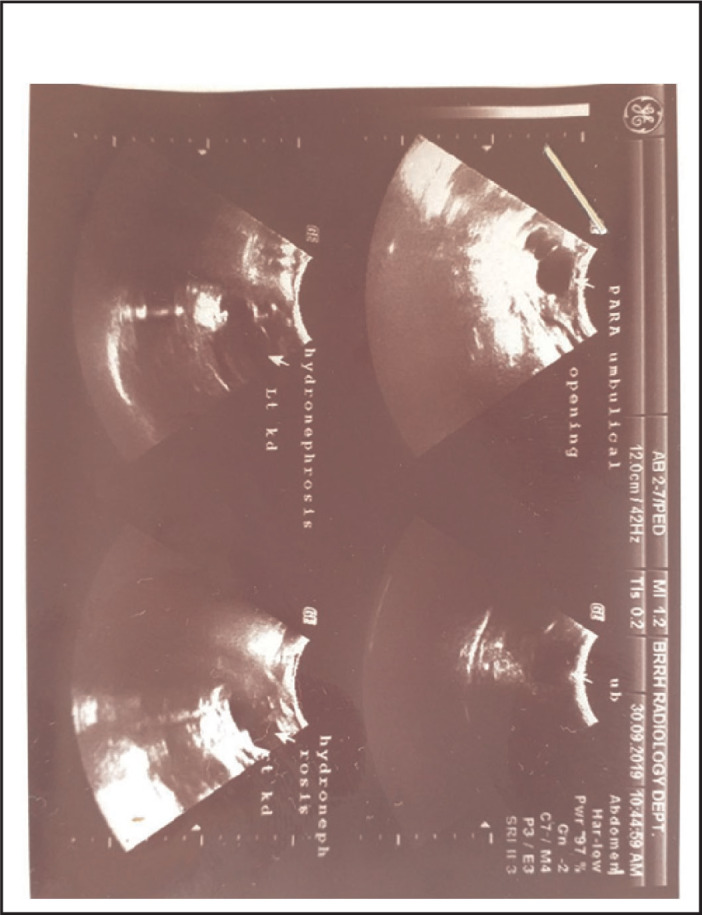


Laboratory investigations (Table 1) revealed increased creatinine level. Urinalysis showed pale yellowish urine, with pus cell 0-2/Hpf, RBCs >100Hpf. His complete blood count was normal. Abdominal ultrasound showed bilateral hydronephrosis (Figure 9).

After investigations, the child was treated with amoxicillin as a case of urinary tract infection, and was referred to urologist at Bugando zonal referral hospital in Mwanza for further management including management of impaired kidney function and possible orchidopexy.

## DISCUSSION

Prune Belly Syndrome (PBS) is a rare congenital disorder composed of anomalies of various organ systems. It is allied with tremendous morbidities as 50% have variable degrees of urinary pathology during their lifetime and 67% develop renal failure^6^. These two case reports describe PBS, one with pectus carinatum and the second case with pectus excavatum. The first case report describes a two months old boy with the triad of PBS as reported in previous publications.^1,4,5^ In the majority of cases the diagnosis of PBS is made during infancy^4^, but with increased use of antenatal ultrasound more cases will be diagnosed and referred to a full facility centre before birth. In both cases no antenatal imaging was performed. Although the child in the first reported case was born at a health facility, his congenital malformation was not detected until 2 months when they were seen at a higher level of health facility (BRRH). This may indicate a low level of awareness among health professionals about PBS. However, there are very few published case reports of diagnosed PBS in East Africa^10^ and we found none in Tanzania. Pectus carinatum is one of the musculoskeletal problems seen in our case (Figure 1) and was also reported in a previous publication^11^, however in the second case pectus excavatum was found (Figure 10). This was also described in previous publication^2^. The characteristics of congenital musculoskeletal problems correlate well with the embryologic theory of PBS^12^. No other dysmorphisms were noted in both cases.

Other documented anomalies which coexist with PBS include cardiopulmonary, gastrointestinal and musculoskeletal anomalies^11^. Currently, there are chances for children with PBS to reach adulthood. However, morbidity related to the cardiopulmonary system including respiratory infection and sepsis can potentially affects their quality of life. The abdominal wall defect may not have life-threatening consequences but the inefficient contraction of the abdominal wall muscles is believed to affect bladder, bowel, and pulmonary function, increasing the risk of recurrent urinary and respiratory tract infections^4^. As was seen in both cases, hydronephrosis was present, with the first case having normal creatinine level and the second case having elevated creatinine. Treatment for the first case was intended to save life by stabilizing the patient first. However, this was not successful despite the antibiotics and care given at our centre. After stabilization, a surgical team is of importance for further management. After communicating with an urologist, **case 1** was referred after correction of anaemia, however, he got septicaemia (clinical diagnosis) and died before being seen by an urologist. In the second case we managed to make the diagnosis early and he was referred to the zonal referral hospital for further management including management for impaired kidney function and orchidopexy. There have been documented cases of testicular tumours developing in PBS patients, but overall, the risk does not appear to be greater than other patients with undescended testes^4^. Orchidopexy or removal of the testes are treatment modalities available to children with PBS. In our cases, this was one of the plans behind the referral to urologist. When timely orchidopexy has not been performed, removal of the testes is done to counteract the high risk of cancer associated with intraperitoneal testes. Our approach of these cases was to give the best treatment and care that was available in our setting. Nevertheless, unavailability of urologist, and of facilities for blood culture, C reactive protein and serum electrolytes in our hospital limited our approach to care for such patients.

## CONCLUSIONS

In resource limited settings, diagnosis of PBS needs a high index of suspicion. In order to carry out the appropriate steps for management and follow up, timely diagnosis of PBS preferably during antenatal stage or shortly after birth is of great importance, especially at peripheral health facilities. Antenatal ultrasound facilities and sensitizing health personal at peripheral antenatal/ obstetric health facilities are crucial for early diagnosis. Since the disease involves multiple organ systems, a multidisciplinary management approach, including psychosocial support, is necessary in order to advance the quality of life for patients with PBS which implies timely referral to a centre with adequate imaging, laboratory, paediatric and surgical/urological facilities. We recommend more training in the pre and postnatal recognition and management of syndromes including PBS for practitioners in lower level facilities. Referral facilities also need to be better staffed with various specialists and better equipped to care and treat PBS patients.

## Abbreviations

BP: : Blood pressure
BRRH: : Bukoba Regional Referral Hospital
CBC: : Complete Blood Count
OPD: : outpatient department
PBS: : Prune belly syndrome
PR: : Pulse rate
RBC: : Red blood cell
RR: : Respiratory rate
RUL: : Right Upper Lobe
SpO_2_: : Stand for Peripheral capillary Oxygen Saturation
T: : Temperature
WBC: : White Blood Count
